# THE HEALTHY BRAIN INITIATIVE ROAD MAP CATALYZES ACTION AMONG STATE, LOCAL, AND NATIONAL PARTNERS

**DOI:** 10.1093/geroni/igad104.0851

**Published:** 2023-12-21

**Authors:** Meghan Fadel, Peter Holtgrave, Carissa Beaty, Shelby Roberts

**Affiliations:** Alzheimer’s Association, Chicago, Illinois, United States; National Association of County and City Health Officials (NACCHO), Washington, District of Columbia, United States; Emory University, Atlanta, Georgia, United States; Alzheimer’s Association, Chicago, Illinois, United States

## Abstract

The Healthy Brain Initiative Road Map Series (Road Map) has achieved measurable success in spurring action in health departments across the country. Evaluation data from the Alzheimer’s Association shows all 50 states have implemented actions from the 2018-2023 Road Map and the majority of states completed at least one action in each of the four domains of the Road Map. The Healthy Brain initiative has also sponsored initiatives to accelerate the implementation of Road Map actions. The projects described are separate from the CDC’s BOLD Program Awards for state and local health departments. The Road Map Strategist Initiative supports local health departments and tribal organizations. It has funded 16 agencies over the past two years to implement Road Map actions in their communities. A coordinated campaign to increase implementation and utilization of the BRFSS caregiving and subjective cognitive decline modules has near nationwide uptake, and, in 2023, a new project, Data for Action, launched in four states to support the use and dissemination of BRFSS and other data. Together, these projects improve assessment, planning and measurement of community health outcomes related to brain health. The 4th edition of the HBI Road Map has an accompanying evaluation tool offering common measures for state and local health departments to track their success as they implement the new actions. This session will explore the past impact of Road Map actions and opportunities for future measurement.

